# The Therapeutic Role of Lamotrigine in Borderline Personality Disorder: A Comprehensive Review of Outcomes, Mechanisms, and Treatment Strategies

**DOI:** 10.7759/cureus.67362

**Published:** 2024-08-21

**Authors:** Rachi M Ade, Pradeep S Patil, Aniket Pathade

**Affiliations:** 1 Clinical Psychology, School of Allied Health Sciences , Datta Meghe Institute of Higher Education and Research, Wardha, IND; 2 Psychiatry, Jawaharlal Nehru Medical College, Datta Meghe Institute of Higher Education and Research, Wardha, IND; 3 Medicine, Jawaharlal Nehru Medical College, Datta Meghe Institute of Higher Education and Research, Wardha, IND

**Keywords:** emotional regulation, mood stabilizers, psychopharmacology, lamotrigine, borderline personality disorder

## Abstract

Borderline personality disorder (BPD) is a severe mental health condition characterized by pervasive instability in mood, self-image, and interpersonal relationships, significantly impacting individuals' personal, social, and occupational functioning. Current treatment strategies primarily include psychotherapy and pharmacotherapy, but there remains a need for more effective and targeted pharmacological options. This review examines the therapeutic role of lamotrigine in BPD, focusing on its efficacy, safety, mechanisms of action, and practical treatment strategies. A comprehensive review of the existing literature was conducted, including clinical trials, observational studies, and relevant pharmacological data. Key focus areas included lamotrigine’s impact on BPD symptoms, pharmacological profile, and comparative effectiveness with other treatments. Lamotrigine has shown promise in managing BPD symptoms, particularly in stabilizing mood and reducing emotional dysregulation and impulsivity. Clinical trials suggest that lamotrigine can effectively address core symptoms of BPD, with a safety profile generally comparable to other treatments. The medication’s mechanism of action, which involves modulation of glutamate release and mood stabilization, aligns with the therapeutic goals for BPD. Lamotrigine represents a potential adjunctive treatment for BPD, offering benefits in mood stabilization and symptom management. Integrating psychotherapy and other pharmacological options should be considered within a multimodal treatment approach. Further research is needed to better understand its long-term efficacy and safety and its role in combination therapy.

## Introduction and background

Borderline personality disorder (BPD) is a multifaceted mental health condition marked by pervasive instability in mood, self-image, and interpersonal relationships. According to the Diagnostic and Statistical Manual of Mental Disorders, Fifth Edition (DSM-5), BPD is characterized by a pattern of instability in interpersonal relationships, self-image,, and affects, alongside marked impulsivity [1]. The disorder is defined by at least five of the following criteria: frantic efforts to avoid abandonment, unstable and intense relationships, identity disturbance, impulsivity, recurrent suicidal behavior, affective instability, chronic feelings of emptiness, intense anger, and transient stress-related paranoid ideation or dissociation. These symptoms contribute to a significant burden, with BPD affecting about 1.6% of the general population and presenting even higher rates among psychiatric patients [2]. The disorder typically emerges in late adolescence or early adulthood, often leading to considerable impairment in personal, social, and occupational functioning. The impact of BPD extends beyond individuals, affecting families, caregivers, and the healthcare system, with high rates of self-harm, suicidal behavior, and comorbid conditions [3].

Current treatment for BPD primarily revolves around psychotherapy, with dialectical behavior therapy (DBT) being one of the most established and widely used approaches. Developed by Dr. Marsha Linehan, DBT integrates cognitive-behavioral techniques with mindfulness and acceptance strategies [4]. It focuses on helping patients develop skills in mindfulness, distress tolerance, emotion regulation, and interpersonal effectiveness. Evidence supports DBT’s efficacy in reducing self-harm behaviors and improving emotional regulation. Other psychotherapeutic modalities, such as mentalization-based therapy (MBT) and transference-focused psychotherapy (TFP), also play significant roles in treating BPD, each addressing different aspects of the disorder [5]. Pharmacotherapy, while not specifically approved for BPD, is often used alongside psychotherapy to address specific symptoms or comorbid conditions. Antidepressants, such as selective serotonin reuptake inhibitors (SSRIs), are commonly prescribed to manage mood instability and depressive symptoms [6]. Mood stabilizers like lithium and valproic acid may help with mood swings and impulsivity, while atypical antipsychotics can be beneficial for symptoms of paranoia or severe mood dysregulation. Anxiolytics may be used for anxiety but are generally avoided due to the risk of dependence. Despite these options, the effectiveness of pharmacological treatments can be variable, highlighting the need for more targeted and effective therapies [7].

In recent years, lamotrigine, a medication traditionally used for epilepsy and bipolar disorder, has gained attention as a potential treatment for BPD. Its mood-stabilizing properties and effects on depressive episodes in bipolar disorder suggest it might also benefit individuals with BPD, particularly in managing mood instability and impulsivity. Lamotrigine's mechanism of action involves modulation of glutamate release and mood stabilization, which aligns with the therapeutic goals in BPD. The emergence of lamotrigine as a treatment option necessitates a thorough review of its efficacy and safety in this context [8]. This review aims to comprehensively evaluate lamotrigine’s role in treating BPD by examining its therapeutic outcomes, underlying mechanisms, and treatment strategies. It will summarize current clinical evidence on lamotrigine’s effectiveness and safety for BPD, explore its pharmacological profile about the disorder, and discuss practical considerations for its use in clinical settings. By synthesizing existing research and clinical insights, this review seeks to guide clinicians in integrating lamotrigine into a multimodal approach to managing BPD.

## Review

Lamotrigine: overview

Lamotrigine is a medication primarily employed as an antiepileptic and mood stabilizer, particularly for treating epilepsy and bipolar disorder. Its unique pharmacological profile underpins its effectiveness in these conditions [9]. The mechanism of action of lamotrigine involves several pathways crucial to its therapeutic effects. It primarily inhibits glutamate excitotoxicity by blocking voltage-gated sodium channels, thereby preventing excessive glutamate release, an excitatory neurotransmitter [10]. This action is pivotal in reducing seizure activity and stabilizing mood in bipolar disorder. Additionally, lamotrigine enhances gamma-aminobutyric acid (GABA) release, an inhibitory neurotransmitter that reduces neuronal excitability. Its dual action on excitatory and inhibitory neurotransmitters contributes to its anticonvulsant and mood-stabilizing effects. Moreover, lamotrigine interacts with various receptors, including calcium channels and adenosine receptors, influencing its overall pharmacological profile [10]. Pharmacokinetically, lamotrigine is rapidly absorbed with an oral bioavailability of approximately 98%. Peak plasma concentrations are typically achieved within 1.4 to 4.8 hours post-administration. The drug is metabolized in the liver via glucuronidation, yielding an inactive metabolite. It has a half-life ranging from 24 to 30 hours, allowing for once or twice daily dosing, which enhances patient adherence. Lamotrigine's volume of distribution is 0.9 to 1.3 L/kg, and it binds to plasma proteins at about 56%, characteristics that contribute to its therapeutic profile [11]. Approved for treating various types of epilepsy, including partial and generalized seizures, lamotrigine is often used adjunctively for patients unresponsive to other antiepileptic drugs. In psychiatry, it serves as an effective mood stabilizer, particularly in preventing depressive episodes in bipolar disorder. Its approval for the maintenance treatment of bipolar I disorder, alongside lithium, represents a significant advance in managing this condition [12]. In addition to its approved uses, lamotrigine has been investigated for off-label applications in psychiatry. It has been explored for managing BPD, with some studies suggesting potential benefits in affective instability. However, its efficacy for BPD remains inconclusive. Lamotrigine also shows promise in treating certain neuropathic pain conditions, though this is still under investigation. It may be considered for other mood-related disorders, but further research is necessary to establish its effectiveness in these areas [13].

Lamotrigine for BPD

The clinical efficacy of lamotrigine in treating BPD has been rigorously examined, particularly through key clinical trials. The most prominent is the Lamotrigine and Borderline Personality Disorder: Investigating Long-term Effectiveness (LABILE) trial, a multicenter, double-blind, placebo-controlled, randomized study conducted between July 2013 and November 2016. This trial enrolled 276 participants aged 18 and older, all diagnosed with BPD. Participants were randomly assigned to receive either lamotrigine (up to 400 mg/day) or a placebo, with follow-up assessments conducted at 12, 24, and 52 weeks. The results were significant, showing that lamotrigine did not significantly improve BPD symptoms compared to placebo. Specifically, there were no notable differences in scores on the Zanarini Rating Scale for Borderline Personality Disorder (ZAN-BPD) or in secondary outcomes such as depressive symptoms, self-harm behaviors, or social functioning [14]. These findings from the LABILE trial stand in stark contrast to earlier, smaller studies that had suggested potential benefits of lamotrigine for managing BPD symptoms, particularly emotional dysregulation. However, the robust evidence from the LABILE trial supports the conclusion that lamotrigine is not an effective treatment for BPD. This aligns with current clinical guidelines, which recommend against the use of medications as primary interventions for BPD, emphasizing the importance of psychotherapy as the cornerstone of treatment [14]. In assessing the effectiveness of lamotrigine in managing the core symptoms of BPD, the LABILE trial demonstrated that it did not significantly alleviate emotional instability, impulsivity, or interpersonal difficulties. Emotional instability, a hallmark of BPD, remained unchanged in participants taking lamotrigine compared to those receiving a placebo [14]. This finding challenges the earlier hypothesis that mood stabilizers could effectively manage this symptom. Similarly, impulsivity, another critical aspect of BPD, did not improve with lamotrigine treatment. The lack of significant changes in impulsivity-related behaviors further supports the conclusion that lamotrigine is ineffective for addressing this aspect of the disorder. Additionally, interpersonal difficulties, which are central to the BPD experience, were not alleviated by lamotrigine, as evidenced by the absence of improvements in social functioning and relationship dynamics [14]. Regarding safety and tolerability, lamotrigine is associated with several common side effects, including dizziness, headache, nausea, and skin rash, which can occur in up to 10% of users. While these side effects are generally manageable, a severe but rare adverse effect is Stevens-Johnson syndrome, a potentially life-threatening skin reaction. Although this risk exists, it can be mitigated through careful dose titration and monitoring for interactions with other medications. Long-term safety data suggest that lamotrigine is generally well-tolerated, particularly in patients with bipolar disorder, where it is commonly used. However, given the lack of efficacy in BPD, the rationale for long-term use in this population is questionable [10]. Clinicians must also be aware of potential drug interactions and contraindications associated with lamotrigine. It can interact with other medications, notably valproate, and oral contraceptives, which can affect its metabolism and increase the risk of adverse effects. Caution is advised when prescribing lamotrigine to individuals with a history of severe skin reactions or those on multiple psychotropic medications [15].

Mechanisms of action in BPD

Lamotrigine has not proven to be an effective treatment for BPD. A large randomized, placebo-controlled trial found no significant difference between lamotrigine and placebo in reducing BPD symptoms, depressive symptoms, self-harm, social functioning, or quality of life over a one-year follow-up period. Although earlier, smaller studies suggested that lamotrigine might be beneficial for affective instability in mild-to-moderate BPD, this more extensive trial provides compelling evidence against its clinical efficacy for the disorder [16]. Current clinical guidelines do not support the use of lamotrigine for BPD, advising against medications as the primary treatment approach. While medications may occasionally help build therapeutic alliances, relieve distress, or treat comorbid conditions in BPD, no drugs are effective as primary treatments. Psychotherapy remains the cornerstone of evidence-based treatment for BPD. Clinicians should exercise caution when prescribing lamotrigine or other medications off-label for BPD in the absence of clear evidence of benefit [17]. Understanding the mechanisms of action in BPD involves examining both neurobiological and behavioral aspects. This comprehensive review explores the neurobiological mechanisms, including neurotransmitter systems and mood regulation, as well as behavioral and psychological mechanisms related to emotional dysregulation and cognitive function [18]. 

BPD is characterized by significant dysregulation of neurotransmitter systems, particularly those involving serotonin, dopamine, and norepinephrine. These neurotransmitters are critical for mood regulation and impulse control. Research suggests that individuals with BPD often exhibit altered serotonin levels, contributing to mood instability and impulsive behaviors. Additionally, dysregulation in dopaminergic pathways may affect reward processing and motivation, complicating emotional responses further [19]. Neuroimaging studies have demonstrated that individuals with BPD often show hyperactivity in the amygdala, a brain region associated with emotional processing, and reduced activity in the prefrontal cortex, which is involved in the cognitive regulation of emotions. This imbalance can lead to difficulties in mood regulation, making individuals more prone to intense emotional responses and impulsive actions. The anterior cingulate cortex (ACC), which plays a role in error detection and emotional regulation, also often shows altered activity in BPD patients [20]. Emotional dysregulation is a core feature of BPD, manifesting as intense emotional responses and difficulty managing these feelings. Behavioral theories propose that individuals with BPD may have heightened emotional sensitivity and reactivity, leading to challenges in effectively regulating emotions. This dysregulation is often exacerbated by environmental factors, such as invalidating experiences during childhood, which can reinforce maladaptive emotional responses [21].

**Figure 1:**
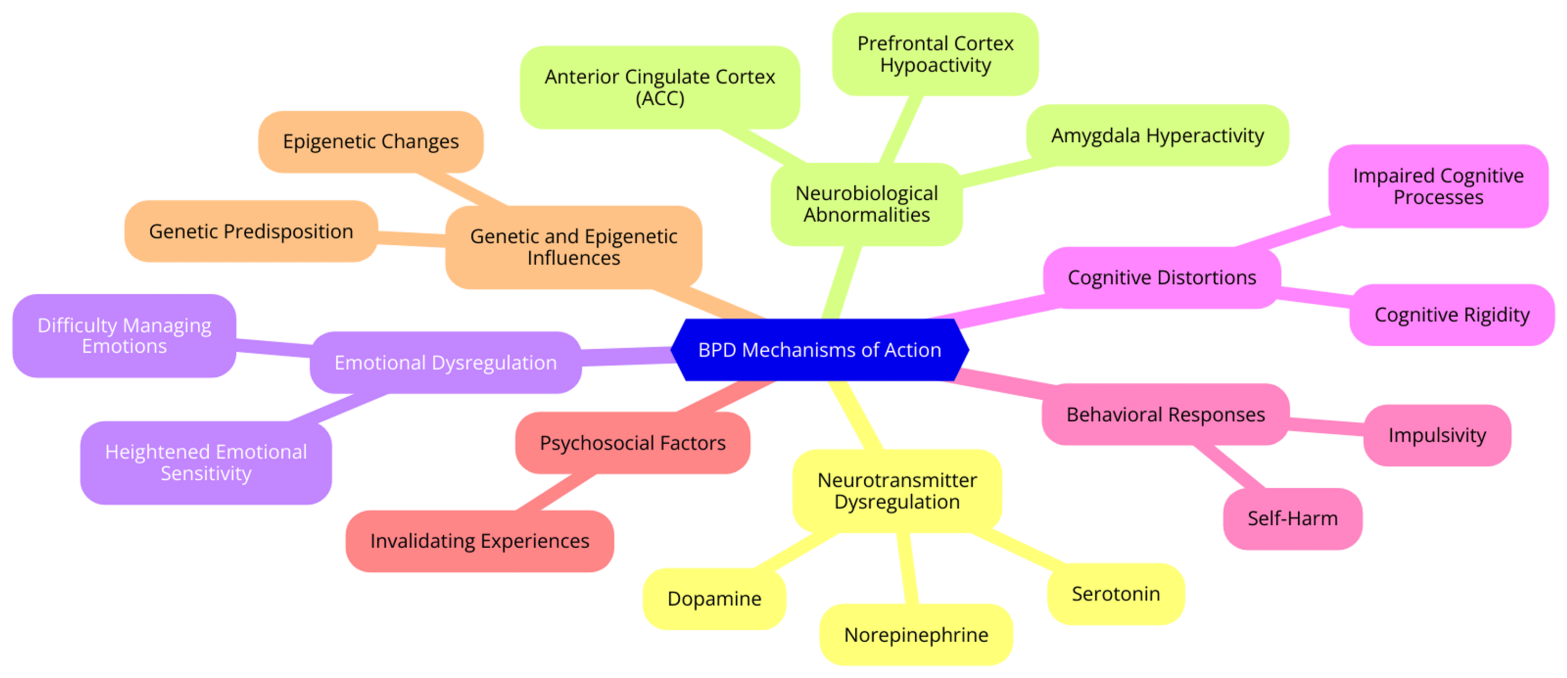
Mechanisms of action involved in BPD BPD: Borderline personality disorder Image credit: Dr. Aniket G. Pathade

Cognitive function in individuals with BPD may also be impaired by emotional dysregulation. Research indicates that emotional states can negatively affect cognitive processes such as attention, memory, and decision-making. For instance, heightened emotional arousal can lead to cognitive distortions, impacting interpersonal relationships and self-perception. Moreover, difficulties in cognitive flexibility may hinder the ability to adapt thoughts and behaviors in response to changing emotional states, perpetuating cycles of emotional distress and maladaptive coping strategies [22]. The mechanisms underlying BPD are complex and multifaceted, involving intricate interactions between neurobiological and psychological factors. A deeper understanding of these mechanisms can inform treatment strategies, highlighting the importance of targeted interventions that address emotional dysregulation and cognitive function. As research continues to advance, it is crucial to integrate findings from neurobiology with behavioral therapies to enhance therapeutic outcomes for individuals with BPD [23]. Mechanisms of action involved in BPD are shown in Figure 1.

Comparative analysis

Lamotrigine has been assessed alongside various pharmacological treatments for mood disorders, including antidepressants, antipsychotics, and other mood stabilizers. Compared to antidepressants, particularly SSRIs such as sertraline and escitalopram, lamotrigine has demonstrated efficacy in alleviating depressive symptoms, significantly outperforming placebo. However, its effectiveness is generally comparable to that of traditional antidepressants [24]. One notable advantage of lamotrigine is its favorable side effect profile; it typically produces fewer adverse effects compared to many SSRIs, which are often associated with issues such as sexual dysfunction and gastrointestinal disturbances. When compared to antipsychotics, lamotrigine has shown similar effectiveness in managing mood symptoms, particularly when compared to atypical antipsychotics like olanzapine. While both classes of medications can be effective, lamotrigine is associated with a lower incidence of metabolic side effects, a common concern with antipsychotic medications. In comparison to other mood stabilizers such as lithium and valproate, lamotrigine has proven effective in stabilizing mood, particularly during the depressive phases of bipolar disorder. However, studies suggest that it does not surpass lithium in overall mood stabilization, making it a preferred option for patients who may not tolerate lithium due to its side effects [25]. Lamotrigine's role in combination therapy is particularly noteworthy. When used alongside psychotherapy, especially DBT, lamotrigine may enhance treatment outcomes for individuals with BPD. By stabilizing mood and reducing emotional dysregulation, lamotrigine enables patients to engage more effectively in therapeutic processes. Additionally, combining lamotrigine with other medications has shown potential benefits [26]. For example, studies indicate that using lamotrigine in conjunction with SSRIs can accelerate the onset of antidepressant effects, making it a valuable option for individuals with treatment-resistant depression. Furthermore, lamotrigine has been found to work well with lithium, enhancing overall mood stabilization without significantly increasing the risk of adverse effects associated with higher doses of either medication [25].

Treatment strategies and considerations

Dosage and Administration

When prescribing lamotrigine, it is crucial to adhere to recommended dosages to ensure both efficacy and safety. For adults with epilepsy, the typical maintenance dose ranges from 300 to 500 mg per day, administered in two divided doses. In the context of bipolar disorder, the target dose is generally around 200 mg per day. A careful dose titration strategy is essential to minimize the risk of adverse effects, particularly skin rashes. Initial doses usually start at 25 mg daily, with gradual increases of 25 to 100 mg every one to two weeks based on the patient's clinical response and concurrent medications [9]. Dose adjustments are particularly critical when lamotrigine is used concurrently with other medications. For example, when co-administered with valproate, a known inhibitor of glucuronidation, the lamotrigine dose may need to be reduced. Conversely, if the patient takes enzyme inducers such as carbamazepine or phenytoin, the lamotrigine dosage may require significant increases, potentially doubling the standard target dose. Careful consideration of these interactions is vital for optimizing treatment outcomes [27].

Patient Selection Criteria

Identifying suitable candidates for lamotrigine therapy is crucial to the treatment process. Patients with epilepsy or bipolar disorder who have not responded adequately to other treatments or who have experienced intolerable side effects may benefit from lamotrigine. Conducting a thorough assessment of the patient's medical history, including previous medication trials and their outcomes, is essential in determining whether lamotrigine is an appropriate option [25]. Special considerations are necessary for certain populations. For older adults with a slower metabolism, dosage adjustments are often required to minimize the risk of adverse effects. Additionally, the safety and efficacy of lamotrigine in children have not been fully established, particularly for those younger than 13 years for bipolar disorder and younger than two years for epilepsy. Therefore, careful monitoring is needed, and alternative treatments may be more appropriate for these age groups [28].

Monitoring and Management

Effective monitoring and management of lamotrigine therapy are crucial for ensuring both patient safety and treatment efficacy. Unlike some medications, routine therapeutic drug monitoring is not recommended for lamotrigine, as dosing should be guided by clinical response and tolerability rather than blood levels. Regular follow-up appointments are essential to evaluate the patient’s progress and make necessary adjustments to the treatment plan [25]. Managing side effects is a key aspect of lamotrigine therapy. Common side effects include dizziness, headache, and rash. A particular concern is the risk of severe skin reactions, such as Stevens-Johnson syndrome, which may occur during the initial titration phase. To mitigate this risk, a gradual titration schedule and vigilant monitoring for any signs of rash are critical. Should a rash develop, lamotrigine should be discontinued immediately and not restarted unless it is confirmed that the rash is unrelated to the medication [29]. For patients who respond well to lamotrigine and tolerate it without significant side effects, long-term management may be beneficial. However, ongoing monitoring remains essential to ensure continued safety and efficacy. Patients should be educated about the signs of potential adverse effects, particularly rashes, and encouraged to report any concerning symptoms promptly. Regular follow-ups will help assess the overall effectiveness of the treatment and allow for adjustments as needed, optimizing the therapeutic benefits of lamotrigine in managing BPD and its associated symptoms [9].

Future directions and research

Recent research has examined the efficacy of lamotrigine in treating BPD. Among the most significant studies is the LABILE trial, which aimed to assess the clinical and cost-effectiveness of lamotrigine compared to a placebo over one year. This trial, involving 276 participants, evaluated a range of outcomes, including BPD symptoms, social functioning, quality of life, and the incidence of suicidal behavior. The findings revealed no significant differences between lamotrigine and placebo in improving BPD symptoms or overall functioning, concluding that lamotrigine may not be a cost-effective treatment for BPD [14]. Despite the ongoing interest in pharmacological treatments for BPD, there remains a substantial need for further research into nonpharmacological interventions. Future studies should focus on the long-term effectiveness of existing treatments, the role of psychotherapy and other nondrug interventions in managing BPD, and the impact of comorbid conditions on treatment outcomes. These areas are crucial for developing comprehensive treatment strategies that address the complex nature of BPD [30]. Research into novel formulations or combinations of existing medications may offer new treatment possibilities. Combining lamotrigine with other mood stabilizers or psychotropic medications could potentially enhance therapeutic outcomes. Additionally, exploring alternative delivery methods, such as extended-release formulations, could improve adherence and efficacy. Such innovations might lead to more personalized treatment approaches, allowing clinicians to tailor therapies to the specific needs of individuals with BPD [31]. Understanding the mechanisms of action of lamotrigine and its effects on mood stabilization is also critical. Emerging research suggests that lamotrigine may modulate glutamate release and affect neuronal excitability, which could play a significant role in emotional regulation. Further investigation into these mechanisms may help identify biomarkers for treatment response and support the development of targeted therapies specifically designed for BPD [32]. Table 1 shows the potential future directions and research areas for the therapeutic role of lamotrigine in BPD.

**Table 1:** Potential future directions and research areas for the therapeutic role of lamotrigine in BPD BPD: Borderline personality disorder; DBT: dialectical behavior therapy

Research area	Description	Potential impact
Long-term efficacy studies	Investigate the long-term effects of lamotrigine on BPD symptoms and overall functioning over extended periods	Provide insight into the sustained benefits and potential risks of long-term use
Combination therapy	Examine the effects of lamotrigine in combination with other pharmacological agents and psychotherapies	Explore how lamotrigine can be integrated into multimodal treatment plans for enhanced outcomes
Mechanisms of action	Delve deeper into how lamotrigine’s modulation of neurotransmitters specifically affects BPD symptoms	Improve understanding of how lamotrigine works in BPD, potentially leading to better-targeted treatments
Comparative effectiveness	Compare lamotrigine directly with other mood stabilizers and antidepressants in treating BPD	Determine whether lamotrigine offers advantages over current standard treatments
Patient subgroup analysis	Study the efficacy and safety of lamotrigine in various demographic subgroups (e.g., age, gender, comorbidities)	Identify specific patient populations that may benefit most from lamotrigine
Safety and tolerability	Conduct research on rare and long-term side effects of lamotrigine, including its impact on quality of life	Ensure that lamotrigine is safe for long-term use and that its benefits outweigh potential risks
Psychotherapy integration	Investigate how lamotrigine can be used alongside specific types of psychotherapy, such as DBT	Optimize the use of lamotrigine within a broader therapeutic framework to enhance overall treatment efficacy
Pharmacogenomics	Explore how genetic variations affect individual responses to lamotrigine	Tailor lamotrigine treatment based on genetic profiles to improve efficacy and reduce side effects
Impact on comorbid conditions	Assess how lamotrigine affects comorbid conditions often seen in BPD, such as depression and anxiety	Determine if lamotrigine can provide additional benefits in managing comorbidities
Innovative formulations	Develop and test new formulations or delivery methods for lamotrigine that may improve patient adherence and outcomes	Enhance the effectiveness and ease of use of lamotrigine in clinical practice

## Conclusions

In conclusion, lamotrigine emerges as a promising treatment option for BPD, offering potential benefits in managing the disorder's core symptoms of mood instability, impulsivity, and emotional dysregulation. Its mood-stabilizing properties, well-documented in the treatment of bipolar disorder and epilepsy, suggest that it may provide significant therapeutic advantages for individuals with BPD, particularly in conjunction with established psychotherapeutic approaches like DBT. While current evidence supports lamotrigine’s efficacy and safety in improving symptomatology and overall functioning in BPD patients, further research is needed to fully understand its role and optimize its use. This includes exploring its effects on diverse patient populations, determining the most effective dosing strategies, and evaluating long-term outcomes. As clinicians consider integrating lamotrigine into their treatment plans, a thorough understanding of its pharmacological profile, potential benefits, and limitations will be crucial for enhancing BPD patient care outcomes.
